# Chemo-Protective Potential of Cerium Oxide Nanoparticles against Fipronil-Induced Oxidative Stress, Apoptosis, Inflammation and Reproductive Dysfunction in Male White Albino Rats

**DOI:** 10.3390/molecules25153479

**Published:** 2020-07-31

**Authors:** Hamida Saleh, Atef M. K. Nassar, Ahmed E. Noreldin, Dalia Samak, Norhan Elshony, Lamiaa Wasef, Yaser H. A. Elewa, Shaimaa M. A. Hassan, Abdullah A. Saati, Helal F. Hetta, Gaber El-Saber Batiha, Masakazu Umezawa, Hazem M. Shaheen, Yasser S. El-Sayed

**Affiliations:** 1 Department of Veterinary Forensic Medicine and Toxicology, Faculty of Veterinary Medicine, Damanhour University, Damanhour 22511, Egypt; Hamida_saleh@vetmed.dmu.edu.eg (H.S.); dalia_samak@vetmed.dmu.edu.eg (D.S.); 2 Pesticides Chemistry and Toxicology, Plant Protection Department, Faculty of Agriculture, Damanhour University, Damanhour, Damanhour 22511, Egypt; atef.nassar@dmu.edu.eg; 3 Department of Histology and Cytology, Faculty of Veterinary Medicine, The Scientific Campus, Damanhour University, Damanhour 22511, Egypt; ahmed.elsayed@damanhour.edu.eg; 4 Department of Pharmacology and Therapeutics, Faculty of Veterinary Medicine, Damanhour University, Damanhour 22511, Egypt; nour1myth@gmail.com (N.E.); lamiaawasef@vetmed.dmu.edu.eg (L.W.); 5 Department of Histology and Cytology, Faculty of Veterinary Medicine, Zagazig University, Zagazig 44519, Egypt; yaserelewa@zu.edu.eg; 6 Laboratory of Anatomy, Department of Biomedical Sciences, Graduate School of Veterinary Medicine, Hokkaido University, Sapporo, Hokkaido 060-0818, Japan; 7 Histology and Cell Biology Department, Faculty of Medicine, Menoufia University, Shebin El-Koom 32511, Egypt; shaimaamer35@yahoo.com; 8 Department of Community Medicine & Pilgrims Healthcare, Faculty of Medicine, Umm Al-Qura University, Makkah 24382, Saudi Arabia; aaasaati@uqu.edu.sa; 9 Department of Medical Microbiology and Immunology, Faculty of Medicine, Assiut University, Assiut 71515, Egypt; helal.hetta@uc.edu; 10 Department of Materials Science and Technology, Faculty of Industrial Science and Technology Soga Laboratory, 9th Floor, Research Labs Building, Tokyo University of Science, 6-3-1 Niijuku, Katsushika, Tokyo 125-8585, Japan; masa-ume@rs.tus.ac.jp

**Keywords:** fipronil, nanoparticles, CeNPs, apoptosis and inflammation gene regulation, male subfertility, reproductive toxicity

## Abstract

Fipronil (FIP) is an insecticide commonly used in many fields, such as agriculture, veterinary medicine, and public health, and recently it has been proposed as a potential endocrine disrupter. The purpose of this study was to inspect the reproductive impacts of FIP and the possible protective effects of cerium nanoparticles (CeNPs) on male albino rats. Rats received FIP (5 mg/kg bwt; 1/20 LD_50_), CeNPs (35 mg/kg bwt) and FIP+CeNPs per os daily for 28 days. Serum testosterone levels, testicular oxidative damage, histopathological and immunohistochemical changes were evaluated. FIP provoked testicular oxidative damage as indicated by decreased serum testosterone (≈60%) and superoxide dismutase (≈50%), glutathione peroxidase activity (≈46.67%) and increased malondialdehyde (≈116.67%) and nitric oxide (≈87.5%) levels in testicular tissues. Furthermore, FIP induced edematous changes and degeneration within the seminiferous tubules, hyperplasia, vacuolations, and apoptosis in the epididymides. In addition, FIP exposure upregulated interleukin-1β (IL-1β), nitric oxide synthase 2 (NOS), caspase-3 (Casp3) and downregulated the Burkitt-cell lymphomas (BCL-2), inhibin B proteins (IBP), and androgen receptor (Ar) mRNA expressions Casp3, nitric oxide synthase (iNOS), ionized calcium-binding adapter molecule 1(IBA1), and IL-1β immunoreactions were increased. Also, reduction of proliferating cell nuclear antigen (PCNA), mouse vasa homologue (MVH), and SOX9 protein reactions were reported. Interestingly, CeNPs diminished the harmful impacts of FIP on testicular tissue by decreasing lipid peroxidation, apoptosis and inflammation and increasing the antioxidant activities. The findings reported herein showed that the CeNPs might serve as a supposedly new and efficient protective agent toward reproductive toxicity caused by the FIP insecticide in white male rats.

Academic Editor(s): Ashok Kakkar

## 1. Introduction

There is a growing interest in the possible negative effects of pesticides exposure on reproduction and fertility [1]. Certain pesticides harm male reproductive capacity by decreasing the sperm count and boosting the incidence of sperm head defects [2]. Fipronil (FIP) is a fairly recent phenylpyrazole insecticide intended for the management of insect resistance problems and global health hazards experienced by older pesticides groups including organophosphate, carbamate, and pyrethroid insecticides [3,4]. It is categorized by world health organization (WHO) as a Class II, relatively toxic, pesticide. It has an acute oral LD_50_ of 97 mg/kg and dermal LD_50_ higher than 2000 mg/kg in rats [5]. It causes extremely harmful effects on aquatic invertebrates, fish, and highland birds, moderate toxicity to mice and rats, while it was not harmful to waterfowl as well as other species of birds [6]. FIP has been categorized as a group C carcinogen depending on increased thyroid follicular cell tumors in both sexes of rat [5,7]. Some pesticides were recorded to have adverse effects on male reproduction [2], including FIP which changes the normal endocrine system function and causes severe reproductive effects [8]. It was postulated that GABA-modulating pesticides, such as FIP, might affect the reproduction of rats at sublethal concentrations [9].

Based on the chemical and physical structure characteristics of nanoparticles, investigations have demonstrated that they can protect cells from damage caused by free radicals [10]. From which, cerium nanoparticle (CeNPs) have attracted much attention in biological applications [11]. CeNPs might protect against cellular damage and radiation caused by toxic substances and under pathological conditions including cardiac or brain ischemia, neurodegeneration of retina, or neurological diseases [12]. Beneficial effects might be efficiently triggered by the regenerative antioxidant properties of CeNPs like scavenging superoxide radicals [13]. CeNPs possess a potent antioxidant activity due to the ratio of Ce^3+^ to Ce^4+^ on their surface and they act by regenerating the endogenous antioxidants [14] i.e., catalase mimetic and superoxide dismutase activity [10,13], nitric oxide and hydroxyl scavenging properties [15]. Unlike dietary antioxidant supplements (e.g., vitamins E and C), CeNPs showed free radical scavenging effect without the need for multiple daily administration [10]. Owing to the ROS scavenging property, CeNPs increased serum testosterone and luteinizing hormone (LH) levels and enhanced sperm quantity and quality [16]. Current research is, therefore, planned to assess the effects of CeNPs as an alternate chemoprotective formula on the quantitative and qualitative levels of sex hormone and tissue apoptosis in male rats.

## 2. Materials and Methods

### 2.1. Experimental Animals and Protocol

Twenty-eight mature male albino rats with 90 ± 10 g body weight were collected from the Animal Breeding Unit, Faculty of Agriculture, Alexandria University (Alexandria, Egypt). Rats were caged in stainless steel metabolic cages under a pathogen-free environment with controlled humidity, temperature (22 ± 2 °C) and a 14 h light/10 h dark cycle. Two weeks before the experiment was conducted, the animals were acclimatized to the testing facility condition to ensure normal behavior and growth. The balanced diet and water were added to rat ad libitum along the experimental period. The animal experiments were carried out based on guidance from the University of Damanhour and approved protocols from the Animal Ethical Committee.

Afterward, the rats were categorized equally into four experimental groups, each consisting of seven rats. Group 1 served as the control group (saline). Group 2—animals orally-received 1 mL of the prepared FIP solution (1/20 LD_50_ ≈ 5 mg/kg bwt; Fiprogent^®^ (80% WG), AgroInvest, Cairo, Egypt) [17]. Group 3—animals orally-received 1 mL of CeNPs solution (1/10 LD_50_ ≈ 35 mg/kg bwt; CeO_2_-NPs < 25 nm, CAS#: 1306-38-3, Sigma-Aldrich Co., St. Louis, MO, USA) [18]. Group 4—animals orally-received FIP+CeNPs at the abovementioned doses. Treatments were done using a gastric tube for 28 consecutive days and the animals observed daily for any clinical signs. On the 29th day, all rats were anesthetized using an anesthesia system containing xylazine: ketamine (1.0:7.5 mg/kg). Immediately, blood samples were collected from the caudal vena cava, then left to coagulate and centrifuged for 15 min at 1390× *g* and the serum was harvested for further biochemical examinations. Immediately after that, the rats were euthanized and dissected, and each rat’s testicles were quickly removed and washed thoroughly with chilled physiological saline. One testicle was collected in liquid nitrogen tank and then frozen at −80 °C for further biochemical and molecular analysis. The other testicle was collected in 4% paraformaldehyde dissolved in phosphate-buffered saline (PBS) for 48 h for further histopathological and immunohistochemical examinations.

### 2.2. Oxidative Stress/Antioxidants Test

A part of the frozen testicle was homogenized in Tris-HCl buffer (50 mM; pH 7.4) (1:9 *w*/*v*) and centrifuged for 30 min at 12,000× *g* and 4 °C. Subsequently, supernatant was used for the colorimetric estimation of tissue malondialdehyde (MDA, lipid peroxide marker) [19], nitric oxide (NO) concentrations [20], superoxide dismutase (SOD) [21], and glutathione peroxidase (GPx) [22] enzymes activity following the manufacturers’ guidelines (Biodiagnostic Co., Cairo, Egypt) using a pgT80 UV-Vis spectrophotometer (PG Instruments Limited, Leicestershire, UK).

### 2.3. Quantitative Reverse Transcription-PCR Test (RT-qPCR)

TOPreal™ RNA Purification Kits (iNtRON Biotechnology, Inc., Seongnam-Si, Korea, Cat. #17211) was used for extracting the total RNA from the frozen testicles section. A 2% agarose gel electrophoresis was used to evaluate the consistency of the extracted RNA. TOPreal™ First Strand cDNA Synthesis Kits (iNtRON Biotechnology, Inc., Seongnam, Gyeonggi, South Korea Cat. #EZ005M) were used to reverse transcribe total RNA to first-strand cDNA. 

The TOPreal™ qPCR 2X PreMIX (SYBR Green with low ROX) (iNtRON Biotechnology, Inc., Cat. #RT500s) and specific primers were used for the detected genes: caspase-3 (Casp3), androgen receptor (Ar), inhibin B protein (IBP), nitric oxide synthase 2 (NOS), B-cell lymphoma 2 (BCL-2), and interleukin-1β (IL-1β). For real-time PCR examination, Mx3005P real-time PCR system was used (Agilent Technologies, Santa Clara, CA, USA). Gene primer sequences were designed using Primer3 and BLAST programs (National Center for Biotechnology Information, Bethesda MD, 20894 USA) (Table 1). The target gene’s Ct values were normalized with Gapdh’s Ct value of the same sample. All samples were analyzed and conducted in duplicate.

### 2.4. Histopathological Assessment

Fixed testicular samples were fixed according to the routine paraffin embedding method that include drying in ascending degrees of ethanol, disinfected in three xylene, and inserting in melted paraffin wax at 65 °C. Then, paraffin blocking of a sagital section of the testes including the head of the epididymis was prepared. Hematoxylin and eosin (H&E) [23] and Periodic acid Schiff (PAS) [24] were used to stain four µm thick sections. Semiquantitative scoring of testicular and epididymal lesions was calculated according to Gibson-Corley et al. [25]. Briefly, lesions in 10 fields were chosen randomly from each slide for each rat and the obtained results were averaged. The lesions were scored in a blinded way (Score scale: 0 = normal, 1 ≤ 25%, 2 = 26–50%, 3 = 51–75%, and 4 = 76–100%). Furthermore, we used Johnsen scoring method of Johnsen for analysis of the changes in the spermatogenesis among the studied groups [26]. Briefly, the H&E stained testicular tissue sections were examined under 20 high power fields and 50 tubular sections/each testis were evaluated and scores were reported according to the Johnsen’s scores criteria shown in Table 2, and the average Johnsen score/group was calculated.

### 2.5. Immunohistochemistry (IHC)

Working dilutions, sources, methods, and antibodies for antigen recovery were listed in Table 3. The immunohistochemical technique described by Noreldin, Elewa, Kon, Warita and Hosaka [27] was used in 4 µm tissue sections. Briefly, endogenous peroxidase enzyme was deactivated by incubation with H_2_O_2_ (3% in absolute methanol) at 4 °C for 30 min and washed again using PBS. Nonspecific reaction at was blocked with 10% normal blocking serum (Sigma-Aldrich, Cat: A9647) for 60 min at room temperature. The primary antibodies were then incubated overnight at 4 °C, washed with PBS, incubated for 60 min with biotin-conjugated goat anti-rabbit IgG antiserum or rabbit anti-goat IgG antiserum (Histofine kit, Nichirei Corporation, Tsukiji, Tokyo, Japan) according to the species’ primary antibody hosted. Then the sections were incubated for 30 min with streptavidin-peroxidase conjugate (Histofine kit) after washing with PBS. The streptavidin-biotin complex was further incubated for 3 min with a solution of 3,3′-diaminobenzidine tetrahydrochloride (DAB)-H_2_O_2_, pH 7.0. Finally, the sections were rinsed with distilled water and stained with Mayer’s hematoxylin counterstain. A digital camera (EC3, Leica, city, Germany) connected to a microscope (Leica DM500, Leica, Germany) was used to capture micrographs of the prepared sections. For the quantification of immunostaining intensities, the Image J software (National Institutes of Health, Bethesda, MD, USA) was used [28]. The inverse mean density of immunopositive area was determined as positive expression as previously reported by Vis, Kranse, Nigg and van der Kwast [29] in 10 randomly chosen fields from various sections of eight rats in each group.

### 2.6. Enzyme-Linked Immunosorbent Assay (ELISA)

Twenty µL serum was firstly incubated with biotinylated monoclonal testosterone specific antibody (Elescys Testosterone, Cobas^®^ Co., Mannheim, Germany). The second incubation was performed after adding microparticles coated with streptavidin and ruthenium complex-labeled testosterone derivatives. The complex was bounded to the solid phase through the reaction between streptavidin and biotin. Afterwards, the reaction mixture was aspired into the measuring cell as the fine particles, which were picked up magnetically on the electrode surface. While ProCell/ProCell M was used to remove the unbound substances. The voltage application to the electrode stimulates the emission of chemiluminescent that was measured by photomultiplier. ELISA assay was done using the 96-well VICAM microplate reader (VICAM, Watertown, MA, USA) with DigiRead Software.

### 2.7. Method and Machine Precision

Precision of the analytical method was confirmed through the repeatability (intra-day assay) and intermediate precision (inter-day assay) [30].The intra-day and inter-day precision of the method were determined by repeating the analysis of a random sample of each assay on same day and over five consecutive days, respectively and results were presented in Table 4 as percentages of coefficient of variation ((mean/standard deviation) × 100).

### 2.8. Statistical Analysis

Data were described as means ± standard errors of means (SEM) and analyzed using Statistical Package for Social Sciences (SPSS), version 20 (SPSS Inc., Chicago, IL, USA) for windows. Results were analyzed statistically by one-way (ANOVA) test. Considerable means have been compared using Tukey’s HSD post hoc test at *p* < 0.05 level.

## 3. Results Sections in Wrong Order–Experimental Is Last–Renumber Everything Affected

### 3.1. Quality Control

The data in Table 4 indicate that the intra- and inter-assay values ranged from 2.50 to 6.53 and 7.91 to 10.12%, respectively. The results of accuracy met acceptable criteria, according to World Health Organization [31].

### 3.2. Antioxidant Status and Lipid Peroxidation in Testicular Tissue and Serum Testosterone Levels

The impacts of FIP intoxication and the CeNPs treatment on testicular oxidative parameters and lipid peroxidation are summarized in Table 4. Compared to control values, rats treated with FIP exhibited significant elevation (*p* ≤ 0.05) in testicular NO and MDA levels by approximately 87.50% and 116.67%, respectively, of control associated with decrease in GPx (50.00% of control) and SOD (46.67%) enzyme activities. Conversely, the FIP+CeNPs group demonstrated reduction in testicular NO and MDA levels accompanied with increased SOD and GPx activities in relation to rats treated with FIP. In addition, the antioxidant biomarkers have been restored in the FIP+CeNPs-treated rats in comparison with the control values. Additionally, as shown in Table 4, in the FIP-treated group, the hormonal analysis showed a decline of 60% of the controls’ value in the testosterone. Furthermore, in the FIP+CeNPs and CeNPs groups, blood testosterone has approximately returned to normal compared to control values.

### 3.3. Gene Expressions in Testicular Tissue

The levels of mRNA expression of Casp3, BCL-2, Ar, IBP, IL-1β, and NOS, were detected using RT-qPCR to assess the activity of CeNPs against reproductive toxicity caused by FIP in mature male rats. FIP caused significant downregulation in the levels of relative mRNA expression of BCL-2, IBP, and Ar and upregulation of Casp3, IL-1β, and NOS genes (Table 5). 

The CeNPs treatment caused downregulation of Casp3 and IL-1β but it caused nothing in the expression of the other tested genes compared to the control. The CeNPs co-therapy affected the expressions of all studied genes; Casp3, IL-1β, and NOS were significantly lower than control. On the other hand, BCL-2 and IBP were greatly upregulated compared to the control while the Ar gene expression was similar to the control.

### 3.4. Histopathological Assessment of Testicular Tissue

**Figure 1:**
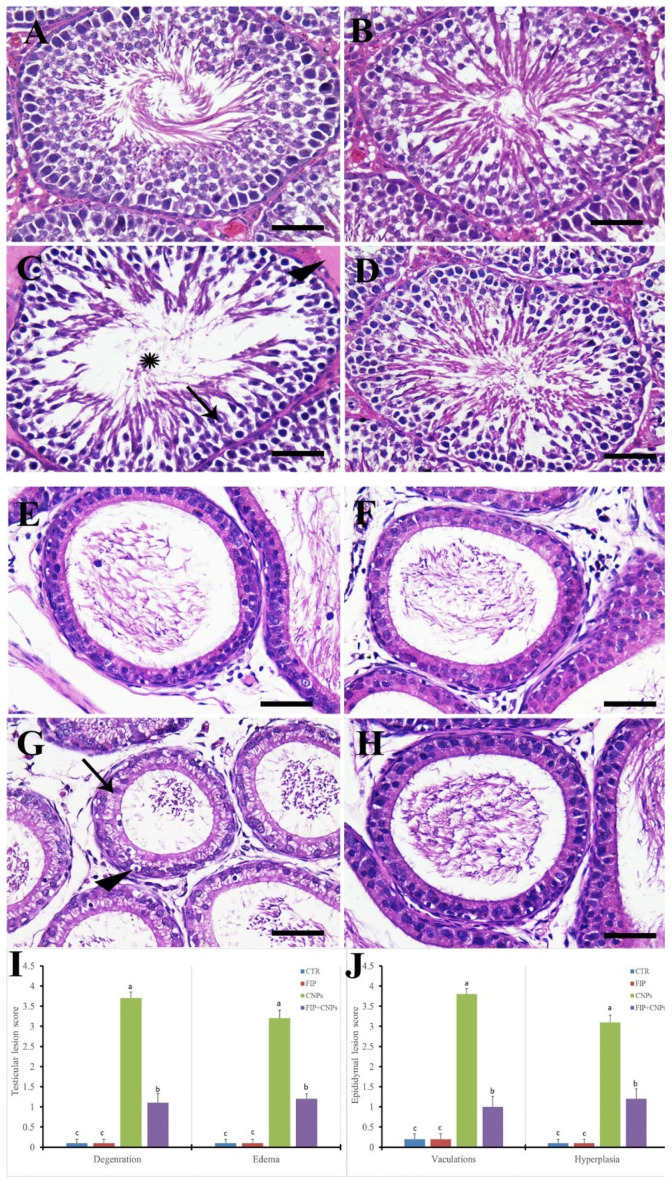
Histopathological examination of rat testis. (**A**) Testicular control, and (**B**) CeNPs groups. (**C**) FIP group revealed edema (arrowhead), degeneration in the different stages of the seminiferous tubules (thin arrow), and fewer spermatozoa in the lumen (asterisk). (**D**) FIP treated with CeNPs. (**E**) Negative epididymal control group. (**F**) CNPs group. (**G**) FIP group showed the phospholipidosis-mediated vacuolations (arrow), hyperplasia and apoptosis of halo cells in the epididymis (arrowhead). (**H**) FIP treated with CeNPs. Scale bar = 50 µm. (**I**) H&E semi-quantitative scores of testicular degeneration and edema. (**J**) H&E semi-quantitative scores of epididymal hyperplasia and vacuolations. Data expressed as mean ± SE, analyzed using one-way ANOVA at *p* ≤ 0.05.

Control and CeNPs groups showed normal testicular architecture consisting of regular, highly organized seminiferous tubules with full spermatogenesis and typical interstitial connective tissue (Figure 1A,B). On the other hand, FIP group revealed edema around the seminiferous tubules, degeneration in the different stages of the spermatogenesis and low number of sperms inside the seminiferous tubules’ lumen compared to the control (Figure 1C). The FIP+CeNPs group showed restoration of the normal architecture of the seminiferous tubules (Figure 1D). In rat epididymis, control and CeNPs groups showed the normal epididymal architecture with normal spermatocytes in the epididymal lumen and normal epithelium (Figure 1E,F). However, FIP group revealed the phospholipidosis mediated vacuolations, hyperplasia, and apoptosis of clear cells in the epididymis (Figure 1G). The FIP+CeNPs group showed amelioration in the epididymal epithelial lesion with more spermatozoa accumulation in the lumen than that of the FIP group (Figure 1H). Semi-quantitative statistical analysis of testicular and epididymal lesions scores declared that the animals treated with FIP had a significantly higher testicular degeneration, edema, epididymal hyperplasia, and vacuolation scores than that of rats in the control and CeNPs groups. However, compared with rats in the FIP group, FIP+CeNPs group showed a significant reduction in the testicular and epididymal lesions score (Figure 1I,J). We analyzed the changes in the spermatogenesis in the testicular tissue among different groups using Johnsen’s score methods as shown in Table 6. Interestingly, the testes of the FIP group showed significant decrease in Johnsen’s score than that of both the Control and CeNPs groups. Furthermore, a significant increase in such score was observed in the testes of FIP+CeNPs group than that of the FIP group. Additionally, the testes of FIP+CeNPs group showed some degree of amelioration where it showed significant increase in the Johnsen’s score than that of the FIP group and a non-significant decrease in the score than the control and CeNPs group.

**Figure 2:**
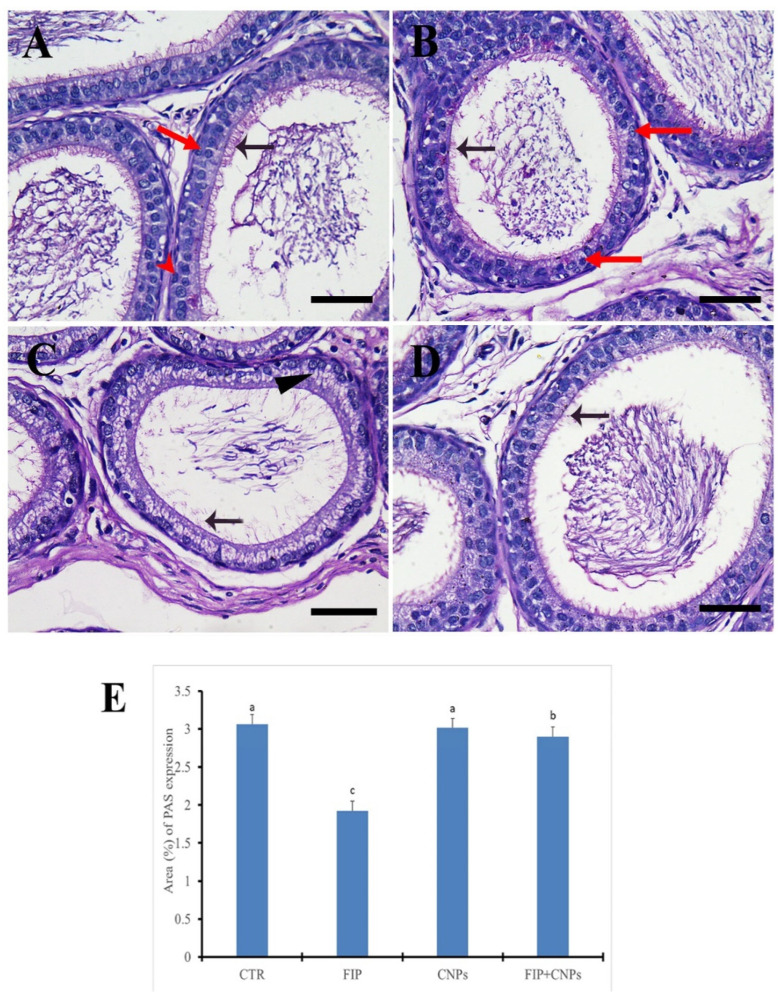
Histochemical staining of rats’ epididymis by Periodic Acid Schiff (PAS). (**A**) Control group showed normal dense short stereocilia (arrow), principal cell (red arrow), basal cell (red arrow). (**B**) CeNPs group. (**C**) FIP group revealed weak PAS reaction in elongated and dispersed stereocilia (arrowhead) with vacuolated epididymal epithelium (arrow). (**D**) FIP treated with CeNPs showed normal dense short stereocilia (arrow). (**E**) Semi-quantification of PAS in the epididymis in different groups. Scale bar = 50 µm. Data expressed as mean ± SE, analyzed using one-way ANOVA at *p* ≤ 0.05.

The PAS-stained epididymis in both control and CeNPs groups showed a normal distribution of PAS positive reaction of the stereocilia that project into the lumen with normal dense and short appearance (Figure 2A,B). However, FIP group revealed a weak PAS reaction of the stereocilia that showed elongated and dispersed appearance and vacuolated epididymal epithelium (Figure 2C). FIP+CeNPs group showed a good protection of epididymal tubules with normal dense short stereo cilia (Figure 2D). Semi-quantitative of PAS distribution of the epididymis revealed normal quantity of PAS reaction in the control and CeNPs groups, but it was reduced significantly in the FIP group. The epidydimal tubules in the FIP+CeNPs treated rats showed significant preservation of the PAS positive reaction for the stereocilia (Figure 2E).

### 3.5. Immunohistochemistry Assessment of Testicular Tissue

**Figure 3:**
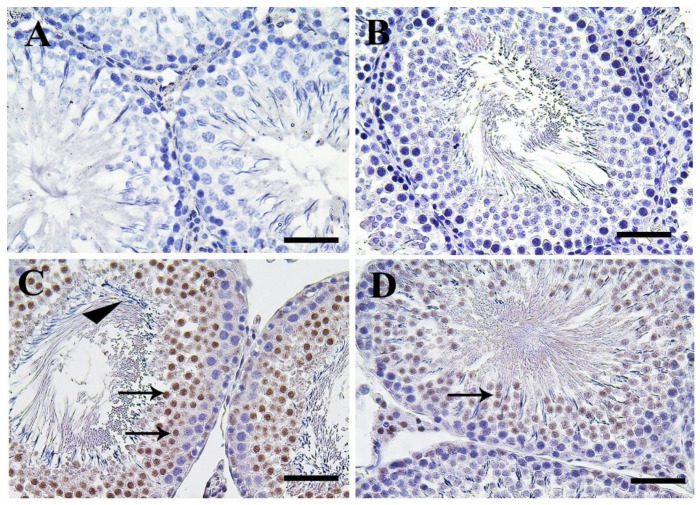

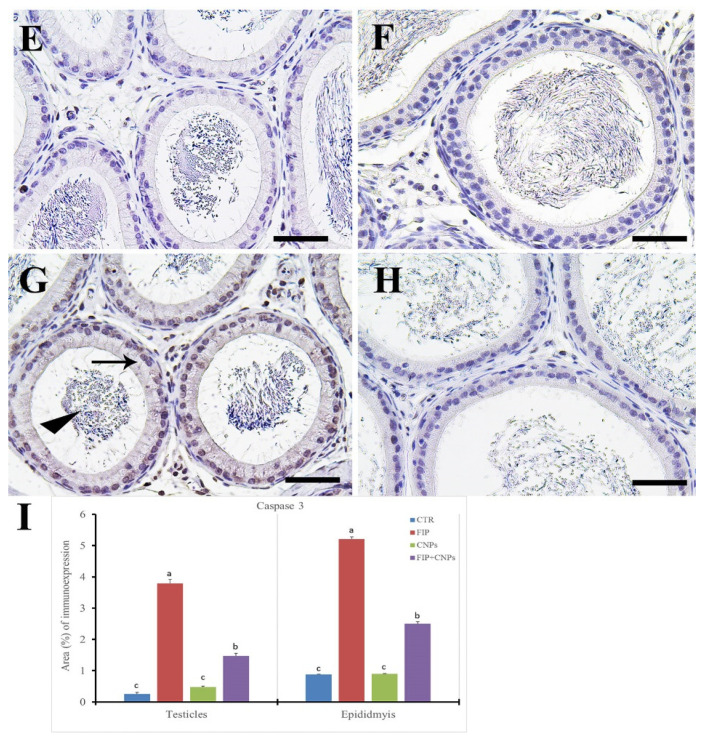
Immunohistochemical staining of rat testis Casp3. (**A**) Control group. (**B**) CeNPs group. (**C**) FIP group revealed strong Casp3 reaction in the nuclei of primary and seconadry spermatocytes (arrows), while all sperms have negative reaction (arrowhead). (**D**) FIP supplemented with CeNPs revealed very weak Casp3 reaction (arrow). (**E**) Epididymal of control group. (**F**) CeNPs group. (**G**) FIP group showed strong positive Casp3 reaction in the nuclei of principal epididymal cells (arrow) and nearly negative reaction in the sperms (arrowhead). (**H**) FIP treated with CeNPs. (**I**) Semi-quantification of Casp3 in the testicular and epididymal tissues in different groups of rats. Scale bar = 50 µm. Data expressed as mean ± SE and analyzed using one-way ANOVA at *p* ≤ 0.05.

Control and CeNPs groups showed a negative Casp3 reaction in seminiferous tubules and interstitial cells (Figure 3A,B). Meanwhile, the FIP group revealed a strong Casp3 reaction in the nuclei of all stages of spermatogenic cells but not for spermatogonia and sperms at the seminiferous tubule’s lumen (Figure 3C). FIP+CeNPs group showed very weak Casp3 reaction in seminiferous tubules epithelium (Figure 3D). In the epididymis, control and CeNPs groups showed a negative Casp3 reaction (Figure 3E,F). However, FIP group showed strong positive Casp3 reaction in the nuclei of principal epididymal cells and nearly negative reaction in the elongated spermatids (Figure 3G). 

**Figure 4:**
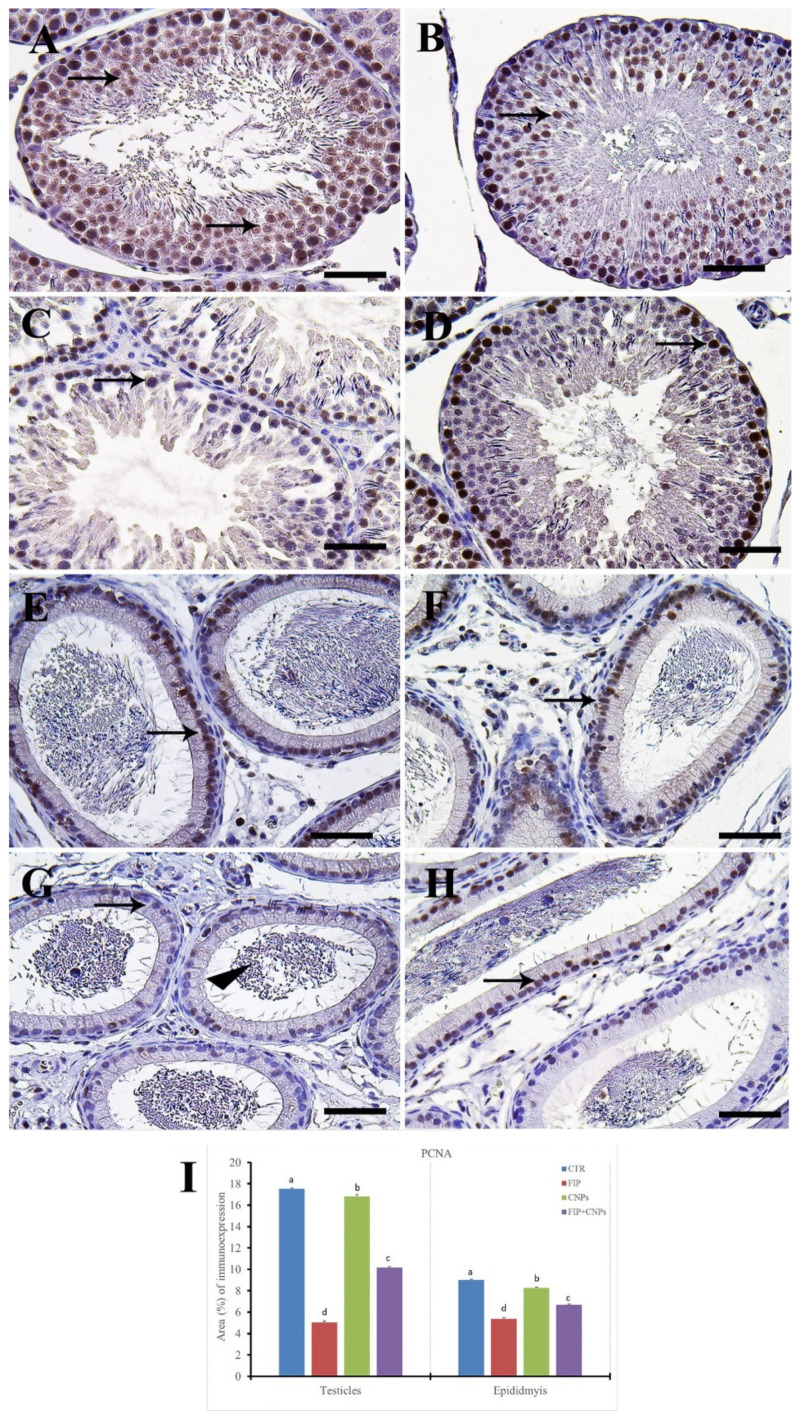
Immunohistochemical staining of rat testis by proliferating cell nuclear antigen (PCNA). (**A**) the control group showed the high PCNA reaction concentrated in the nuclei of all spermatogenic cells (arrows). (**B**) CeNPs group. (**C**) FIP group showed low PCNA reaction (arrows). (**D**) FIP treated with CeNPs. (**E**) Epididymal negative control group showed high PCNA reaction (arrows). (**F**) CeNPs group. (**G**) FIP group with weak PCNA reaction in the epididymal epithelium nuclei (arrow). (**H**) FIP treated with CeNPs. (**I**) Semi-quantification of PCNA in the testicular and epididymal tissues in different groups. Scale bar = 50 µm. Data expressed as mean ± SE, analyzed using one-way ANOVA at *p* ≤ 0.05.

FIP+CeNPs group revealed nearly negative Casp3 reaction in the epididymal tubules (Figure 3H). Semi-quantitative data of Casp3 distribution in the testis and epididymis revealed a significant higher immunolabeling of activated Casp3 protein in the FIP group compared to control and CeNPs-treated rats. This high immune reaction was significantly lowered in the FIP+CeNPs-treated rats (Figure 3I). Immunohistochemical staining of the testicular tissue against the proliferating cell nuclear antigen (PCNA) revealed high reaction in the nuclei of the different stages of spermatogenic cells of control and CeNPs groups with intense reaction in spermatogonia and no reaction in spermatids (Figure 4A,B). On the other hand, FIP group showed weak PCNA reaction restricted in the spermatogonia at the base of seminiferous tubules, while all sperms at the seminiferous tubule’s lumen showed negative reactions (Figure 4C). FIP group treated with CeNPs revealed normal PCNA reaction in all stages of seminiferous tubules epithelium (Figure 4D). Negative control and CeNPs groups showed massive PCNA reaction in all epididymal epithelium (Figure 4E,F). While FIP group showed weak positive PCNA reaction in the epididymal epithelium nuclei (Figure 4G). FIP+CeNPs group revealed nearly strong PCNA reaction in the epididymal tubules (Figure 4H). Semi-quantitative statistical analysis of PCNA distribution in the testis and epididymis revealed a significant lower expression of PCNA in the FIP compared to control and CeNPs-treated rats. This low expression significantly elevated in the FIP+CeNPs treated rats. Furthermore, the the FIP+CeNPs treated rats showed significantly elevated in the PCNA expression than that in the FIP group (Figure 4I).

**Figure 5:**
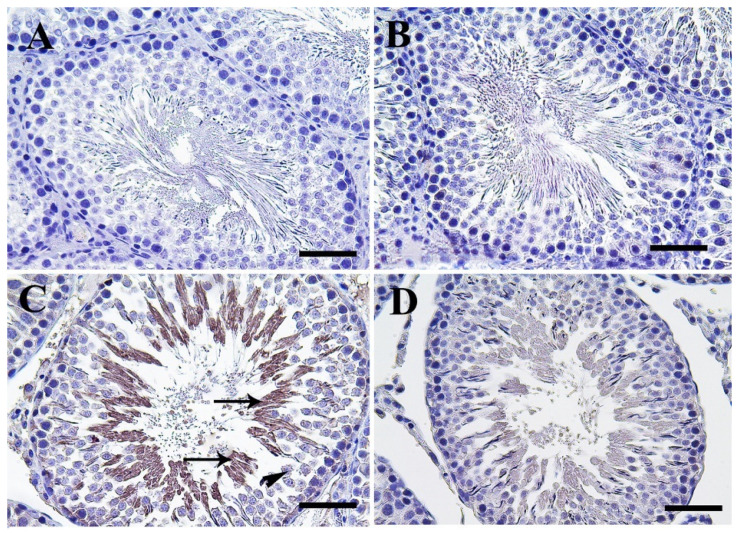

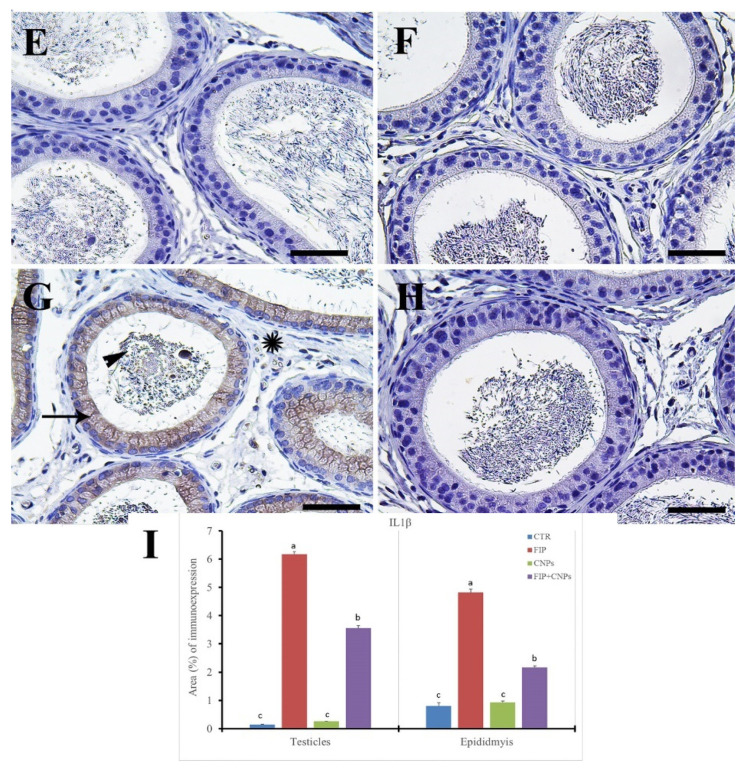
Immunohistochemical staining of rat testes by Interleukin-1β (IL-1β). (**A**) control group. (**B**) CeNPs group. (**C**) FIP group showed strong IL-1β reaction in sperms (arrows), while the remaining spermatogenic cells revealed negative reactions (arrowhead). (**D**) FIP-treated with CeNPs showed very weak IL-1β reaction (arrow). (**E**) Epididymal of control group. (**F**) CeNPs group. (**G**) FIP group showed strong positive IL-1β reaction (arrow) and faint to negative reaction in the spermatozoa (arrowhead) and interstitial connective tissue (asterisk). (**H**) FIP-treated with CeNPs. (**I**) Semi-quantification of IL-1β in the testicular and epididymal tissues in different groups. Scale bar = 50 µm. Data expressed as mean ± SE, analyzed using one-way ANOVA at *p* ≤ 0.05.

Immunohistochemical staining of rat testes by IL-1β showed negative reaction in seminiferous tubules and Leydig cells in control and CeNPs groups (Figure 5A,B). On the other hand, FIP revealed a strong IL-1β reaction in the elongated spermatids, while the remaining spermatogenic cells had negative reactions (Figure 5C). FIP+CeNPs group showed very weak IL-1β reaction in the epithelium of seminiferous tubules (Figure 5D). In epididymis, control and CeNPs groups showed negative IL-1β reaction (Figure 5E,F). Meanwhile, FIP group revealed a strong positive IL-1β reaction in the epididymal epithelial cytoplasm and weak positive to negative reaction in the spermatozoa and interstitial connective tissue (Figure 5G). FIP+CeNPs group showed negative IL-1β reaction in the epididymal tubules (Figure 5H). Semi-quantitative statistical analysis of IL-1β distribution in the testes and epididymis revealed a significant higher expression of IL-1β in the FIP compared to control and CeNPs rats. This high expression was significantly decreased in the FIP+CeNPs treated rats (Figure 5I).

**Figure 6:**
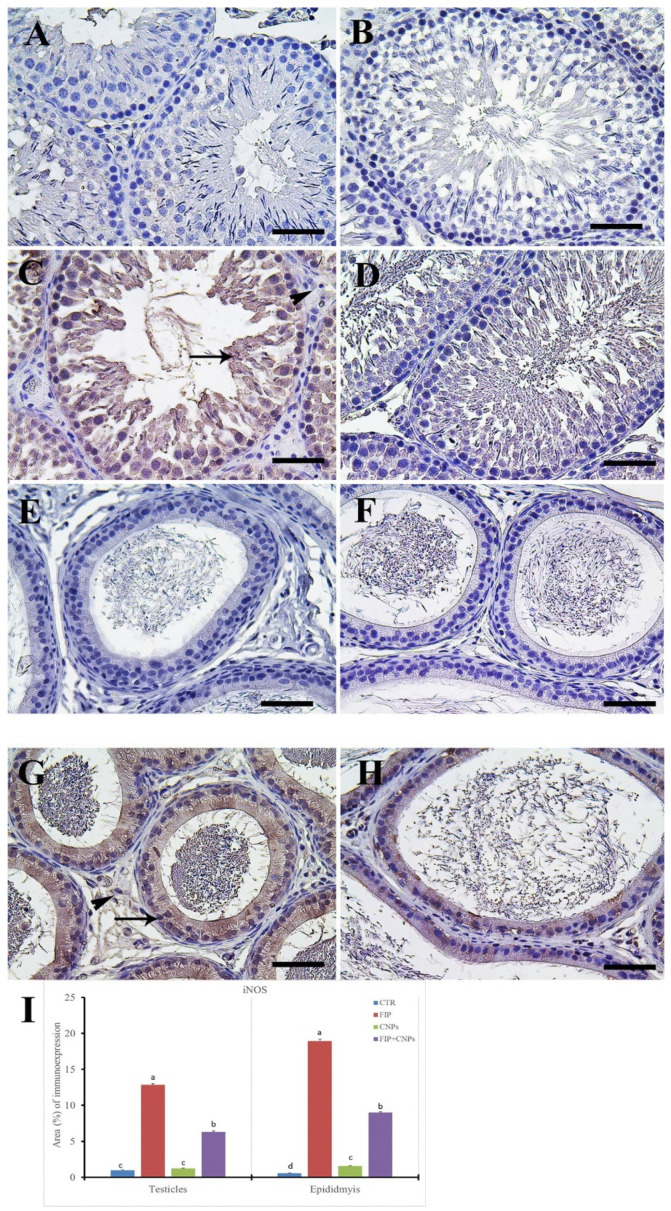
Immunohistochemical staining of rat testis by inducible nitric oxide synthase (iNOS). (**A**) control. (**B**) CeNPs group. (**C**) FIP group showed strong iNOS in the cytoplasm of spermatogenic lineage of the seminiferous tubules (arrow), while the cytoplasm of Leydig’s cells were negatively reacted (arrowhead). (**D**) FIP-supplemented with CeNPs. (**E**) Epididymal control group. (**F**) CeNPs group. (**G**) FIP group showed strong positive iNOS reaction in the epididymal epithelial cytoplasm (arrow) and nearly negative reaction in the interstitial connective tissue (arrowhead). (**H**) FIP-treated with CeNPs. (**I**) Semi-quantification of iNOS in the testicular and epididymal tissues in different groups. Scale bar = 50 µm. Data expressed as mean ± SE, analyzed using one-way ANOVA at *p* ≤ 0.05.

Additionally, both the control and CeNPs groups showed a negative against iNOS reaction in seminiferous tubules cells (Figure 6A,B). However, FIP group revealed a strong iNOS in seminiferous tubules, while Leydig’s cells had negative reactions (Figure 6C). FIP+CeNPs showed a negative iNOS reaction in seminiferous tubules epithelium (Figure 6D). In the epididymis, control and CeNPs groups showed negative iNOS reactions (Figure 6E,F). On the other hand, FIP revealed a strong positive iNOS reaction in the epididymal epithelial cytoplasm and nearly negative reaction in the interstitial connective tissue (Figure 6G). FIP+CeNPs group showed a negative iNOS reaction in the epididymal tubules (Figure 6H). Semi-quantitative statistical analysis of iNOS distribution in the testes and epididymis revealed a significant higher expression of iNOS in the FIP compared to control and CeNPs rats. This high expression significantly decreased in the FIP+CeNPs-treated rats (Figure 6I).

**Figure 7:**
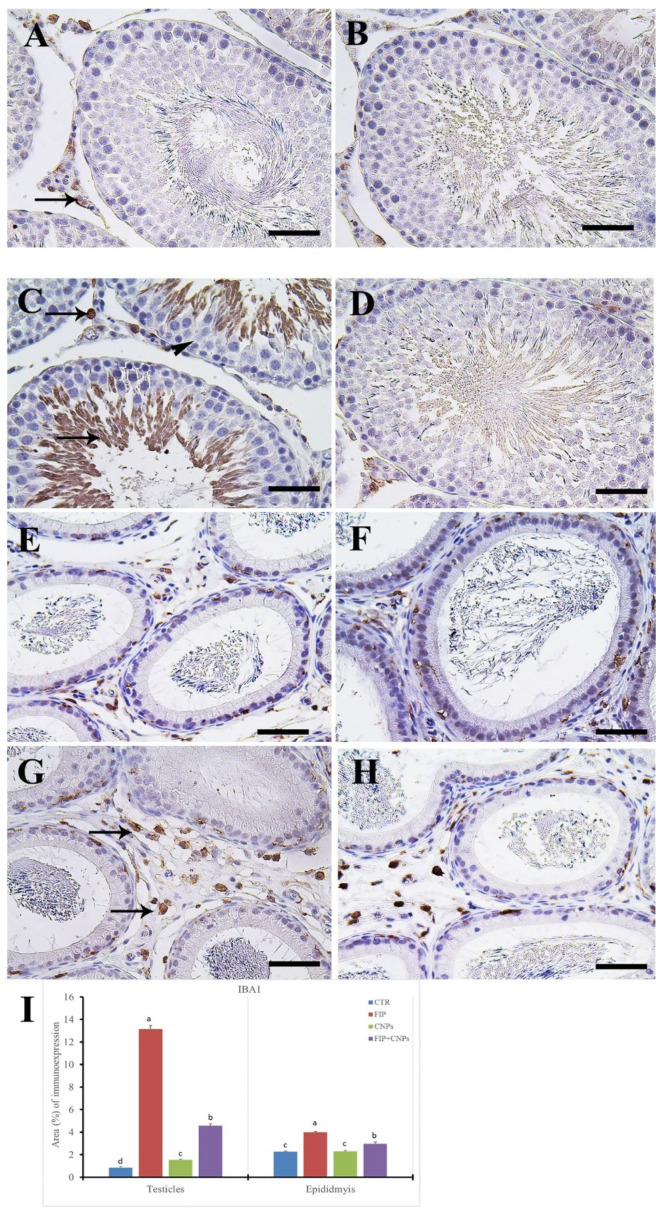
Immunohistochemical staining of rat testis by ionized calcium-binding adapter molecule 1 (IBA1). (**A**) Control group. (**B**) CeNPs group. (**C**) FIP group showed strong IBA1 in most sperms and interstitial tissue (arrows), while all spermatogenic cells revealed negative reactions (arrowhead). (**D**) FIP treated with CeNPs. (**E**) Epididymal of control group showing the negative IBA1 reaction in all epididymal tubules but localized moderately stained in the macrophages in the interstitial connective tissue (arrows). (**F**) CeNPs group. (**G**) FIP group showing dense strong positive IBA1 reaction in the many macrophages in the interstitial tissue (arrows). (**H**) FIP-treated with CeNPs showed low IBA1 macrophages in the interstitial tissue (arrowhead). (**I**) Semi-quantification of IBA1 in the testicular and epididymal tissues in different groups. Scale bar = 50 µm. Data expressed as Mean ± SE, analyzed using one-way ANOVA at *p* ≤ 0.05.

Immunohistochemical staining of rat testis by ionized calcium-binding adapter molecule 1(IBA1) revealed negative reaction in seminiferous tubules in the control and CeNPs groups with few positive cells in the interstitial tissue (Figure 7A,B). Meanwhile, the FIP group showed strong IBA1 reaction in most sperms and interstitial tissue, with negative reactions in spermatogenic cells (Figure 7C). 

The FIP+CeNPs group revealed negative IBA1 reaction in seminiferous tubules epithelium and some positive cells in the interstitial tissue (Figure 7D). Control and CeNPs groups showed negative IBA1 reaction in epididymal tubules but localized moderately in the macrophages present in the interstitial connective tissue (Figure 7E,F). However, FIP group showed dense strong positive IBA1 reaction in many macrophages present in the interstitial tissue (Figure 7G). FIP+CeNPs group showed low IBA1 macrophages present in the interstitial tissue (Figure 7H). Semi-quantitative statistical analysis of IBA1 distribution in the testes and epididymis revealed a significant higher expression of IBA1 in the FIP compared to control and CeNPs-treated rats. This high expression significantly decreased in the FIP+CeNPs treated rats (Figure 7I).

**Figure 8:**
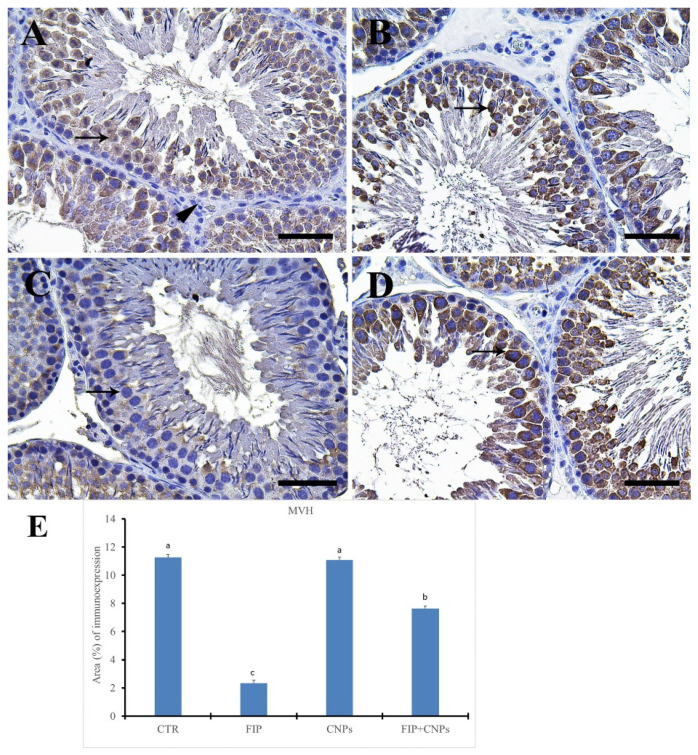
Immunohistochemical staining of rat testis by mouse vasa homologue (MVH). (**A**) Control group showed the strong positive MVH reaction in all spermatogenic cells (arrow) with negative reaction in the interstitial tissue (arrowhead). (**B**) CeNPs group. (**C**) FIP group revealed nearly negative MVH in all spermatogenic cells (arrow). (**D**) FIP treated with CeNPs showing strong positive MVH reaction in all spermatogenic cells (arrow). (**E**) Quantification of MVH in the testicular tissues in different groups. Scale bar = 50 µm. Data expressed as mean ± SE, analyzed using one-way ANOVA at *p* ≤ 0.05.

Immunohistochemical staining of control and CeNPs groups by mouse vasa homologue (MVH) revealed a strong positive reaction in spermatogenic cells with negative reaction in the interstitial tissue of rat testis (Figure 8A,B). On the other hand, FIP showed nearly negative MVH in spermatogenic cells (Figure 8C). FIP+CeNPs group revealed strong positive MVH reaction in spermatogenic cells (Figure 8D). Semi-quantitative statistical analysis of MVH distribution in the testis revealed a significant lower expression of MVH in the FIP compared to control and CeNPs rats. This low expression significantly increased in the FIP+CeNPs treated rats (Figure 8E).

**Figure 9:**
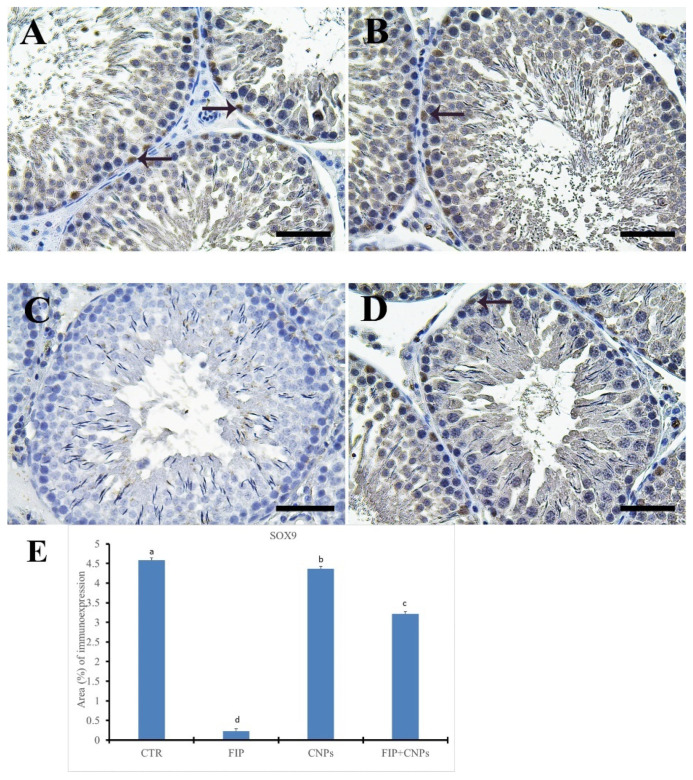
Immunohistochemical staining of rat testes by Sox9. (**A**) control group showed the strong positive Sox9 reaction in Sertoli cells (arrows). (**B**) CeNPs group. (**C**) FIP group showing negative Sox9 in Sertoli cells. (**D**) FIP treated with CeNPs showed strong positive Sox9 reaction in Sertoli cells (arrows). (**E**) Semi-quantification of Sox9 in the testicular tissues in different groups. Scale bar = 50 µm. Data expressed as mean ± SE, analyzed using one-way ANOVA at *p* ≤ 0.05.

Control and CeNPs groups showed a strong positive Sox9 reaction in Sertoli cells (Figure 9A,B). However, FIP group revealed negative Sox9 in Sertoli cells (Figure 9C). FIP+CeNPs group showed strong positive Sox9 reaction in Sertoli cells (Figure 9D). Semi-quantitative statistical analysis of Sox9 distribution in the testis revealed a significant lower expression of Sox9 in the FIP compared to control and CeNPs rats. This low expression significantly increased in the FIP+CeNPs treated rats (Figure 9E).

## 4. Discussion

In this study, the male reproductive toxicity of FIP and the protective role of CeNPs administration against FIP tissue damage were assessed. Administration of 5 mg/kg/day of FIP for 28 successive days to male albino rats resulted in subacute testicular toxicity. FIP administration caused oxidative damages in the testicular tissues, as demonstrated by increased levels of MDA and NO tissue with reduced activity of antioxidant enzymes such as GPx and SOD [32]. Exposure to xenobiotics including pesticides results in increased production of ROS with a recurrent cellular damage of proteins, DNA, and lipids [33,34]. Furthermore, ROS could influence the NO’s bioavailability from its biosynthesis to complete removal [35]. Nitric oxide is a highly-reactive free radical, which persuades nitrosative stress by targeting DNA, lipids, and proteins, besides it remarkably affect the antioxidant defense mechanisms [36].

On the contrary, the endogenous antioxidants i.e., GPx, and SOD effectively eliminate free radicals and protect cells from the oxidative damage [37]. It was possible to reduce the antioxidant effects of FIP, which were revealed herein due to their excessive consumption in suppressing free radicals generated throughout the metabolism of FIP. The FIP impacts might be because of its capacity to increase the production of NOS and ROS, and LPO, which might harm the biological membranes in the testicular tissues. This might lead to Leydig and spermatogenic cells degeneration that interferes with spermatogenesis. Moreover, the decrease in testosterone levels in the serum clearly illustrated the inhibitory effect of FIP on the pituitary gonadotropins (FSH and LH) secretion and thus on the biosynthesis of testosterone, which arrests spermatogenesis [38]. Moreover, the subacute exposure of FIP stimulated histopathological alterations in the seminiferous tubules like edema around the seminiferous tubules, degeneration in the different stages of the seminiferous tubules, and low number of sperms inside the seminiferous tubule’s lumen.

Additionally, FIP has been found to increase the expression level of IL-1β genes, which are considered as a central mediator of the inflammation process and stimulator of the differentiation and proliferation of many cell types. It has been demonstrated that the Sertoli cells constitutively synthesize IL-1β, where this cytokine seems to be included in regulating spermatogenesis [39]. Confirming this, there was a strong reaction of IL-1β in most sperms in FIP-treated group of rats. In addition, the overproduction of ROS due to FIP administration, might led to apoptosis in the testicular tissues mainly in spermatogenic cells, which was evidenced by down regulation in the level of relative BCL-2 mRNA expression and up regulation of Casp3 genes. Apoptosis is either a programmed cell death of self-destruction directed by genes where cells die randomly in a controlled way or it might be triggered by different environmental chemicals or catalysts. ROS and oxidative stress have been associated with apoptosis in different kinds of cell such as spermatogenic cells [40]. Mitochondria is an oxidative stress sensor and its membrane potential loss (mV) or its damage might result in death of the cell by cytochrome *C* and other pro-apoptotic factors release into the cytosol that eventually lead to apoptosomes formation and caspase cascade activation [41].

After rats were exposed to FIP, the percentage of apoptotic sperm has increased markedly, suggesting that FIP might induce programmed cell death in vivo that could be mediated by an oxidative stress mechanism [42]. The BCL-2 proteins are a family of proteins that work in the mitochondrial membrane. They work either for stability like anti-apoptotic (e.g., BCL-2, BCL-XL), which can delay the release of cytochrome *C* or destabilization of the mitochondrial membrane through facilitating the release of cytochrome *C* like pro-apoptotic (e.g., Bax, Bak) by preventing the cytoprotective effects of BCL-2 and BCL-XL [43]. In turn the increased release of cytochrome *C* in the cytosol triggers the activation of caspases 9 and 3 [44]. Caspase3 activation breaks down the DNA, reduces the proliferation of cytoplasmic chromatin, expands the endoplasmic reticulum [45], and finally leads to cell apoptosis [46]. This was confirmed through the effects of FIP, which revealed a strong Casp3 reaction in all stages of spermatogenic cells in testicles and the strong positive Casp3 reaction in the epididymal epithelial nuclei in epididymis by immune histochemical staining.

Moreover, there was downregulation of inhibin B protein gene, which is secreted and synthesized from Sertoli cells. It’s a hormonal protein, inhibin B that specifically regulates follicle-stimulating hormone (FSH) secretion through the anterior pituitary gland [47]. Previous in vitro and in vivo studies failed to detect the relationship among the inhibin levels, Sertoli cell function, and spermatogenesis process [48]. Now the reason for this has become clear with the discovery of two major types of inhibin, namely inhibin A and inhibin B [47]. Inhibin B is the main type developed in males of rats and humans [47,49]; the Sertoli cell thought to be the primary source of circulating inhibin B. Moreover, FIP acts as an Ar antagonists, directly competing for Ar with testosterone and dihydrotestosterone [50] resulting in a decrease in testosterone production. Epididymis relies on androgens to retain the normal epithelial function and architecture, which is essential for normal sperm maturation and transport. Therefore, if androgen stimulation is reduced, the epididymis will atrophy [51]. Continued exposure to the toxic chemicals which reduces testosterone may lead to some level of histologic changes in the androgen-dependent epididymis and this was noticed in rats intoxicated with FIP. Phospholipidosis-mediated vacuolations, hyperplasia and apoptosis of clear cells in the epididymis by H&E stain were noticed. Furthermore, the staining of the rat epididymides by Periodic acid Schiff (PAS) showed that a weak PAS reaction in degenerated stereocilia and vacuolated epididymal epithelium.

On the other hand, a weak PCNA reaction occurred in FIP group was observed in all spermatogonia, with negative reactions to the nuclei of the elongated spermatids. PCNA is effective for germinal arrest diagnosis as reduced levels of PCNA in germinal arrest indicate a decline in DNA synthesis [52]. As well, IBA-1 is a calcium-binding protein composed of 147 amino acids, which resembles in its structure three other calcium-binding proteins: S-100 protein, calmodulin, and troponin C [53]. The IBA-1 protein is considered to be similar to proteins defined by other researchers, such as MRF-1 (microglia response factor-1 [54]), AIF-1 (allograft inflammatory factor-1 [55]), and daintain [56]. Owing to IBA1 protein up-regulation in activated microglia in response to nerve damage, it has been hypothesized that IBA1 might have a role in recovering from brain damages [57]. By using the IBA1 protein-specific antibody we found that IBA1 protein was primarily presented in elongate spermatids cytoplasm [58], neither spermatozoa, spermatogonia, mature epididymal spermatozoa, nor protein-expressed round spermatids [58]. During the mammalian spermatogenesis course, differentiated spermatids still in direct contact with Sertoli somatic cells till spermiation, where fully differentiated spermatids are isolated from their excess cytoplasm and released into the seminiferous tubule’s lumen. IBA1 protein is specifically presented in the elongate spermatid’s cytoplasm, and it is hypothesized that IBA1 may be included in the signal for excess spermatid cytoplasm elimination. We assume that the mRNA of IBA1 might be subjected to regulate posttranscriptional in spermatids and that the protein is exclusively presented and works in spermatids elongation. This protein is located in the cell nucleus and cytoplasm. IBA1 found in the cytoplasm possesses actin-crosslinking effect [59] and is crucially included in some forms of actin cytoskeleton rearrangement related to motility e.g., in the building of phagocytic cups (initial stage of phagocytosis) and in membrane ruffling [60]. Since IBA1 studies concentrated on its function in microglia, little has been reported concerning cell expressing IBA1 types outside the central nervous system (CNS). Macrophages (MΦs) in rat liver and spleen exhibit immunoreactivity in IBA1 [57].

Moreover, there was reaction in most spermatids and interstitial tissue, while all spermatogenic cells in FIP group showed dense strong positive IBA1 reaction in the many macrophages in the interstitial tissue in epididymis. Sox9 is the encoding gene of the steroidogenic factor 1 [61], however it appears to be crucial for Sertoli cell as well as male differentiation. This means that the synthesis of individual factors in Sertoli cell development in different species can be achieved through alternative regulatory mechanisms. As a result, FIP exhibited negative Sox9 in Sertoli cells. MVH protein has been shown to be expressed exclusively in germ cells from E11.5 to the post-meiotic phase in males as well as females [62,63]. Therefore, in the current research, MVH immunohistochemistry was used to assess the effect of FIP on testicular and we found that it showed negative MVH in spermatogenic cells so that the spermatogenesis process could be stopped.

## 5. Conclusions

Current results stated that FIP caused negative impacts on male reproductive functions in rat testis (oxidative stress, inflammation, and apoptosis). Therefore, it might be concluded that this insecticide (FIP) is highly toxic to reproductive function and might alter the fertility of animals. Also, after rats were exposed to FIP, the apoptotic sperm percentage has increased markedly, suggesting that FIP causes programmed cell death in vivo that could be mediated by mechanism associated with oxidative stress. In addition, CeNPs are known to be a recent and effective preventive agent for reproductive toxicity caused by FIP in rat males.

## Figures and Tables

**Table 1:** Sequences of primers used for real time PCR investigations of caspase-3 (Casp3), androgen receptor (Ar), inhibin B protein (IBP), nitric oxide synthase 2 (NOS), B-cell lymphoma 2 (BCL-2), and interleukin-1β (IL-1β) genes.

Gene	Gene Description	Accession Number	Sequences (5′ > 3′)
IBP	Inhibin subunit beta B (IBP), mRNA	NM_080771.1	F: GGGTCCGCCTGTACTTCTTC R: CACCTTGACCCGTACCTTCC
Ar	Androgen receptor (Ar), mRNA	NM_012502.1	F: CAGGGACCACGTTTTACCCA R:TTTCCGGAGACGACACGATG
Caspase-3	Cysteine aspartate specific protease-3	NM_001284409.1	F: AGTTGGACCCACCTTGTGAG R: AGTCTGCAGCTCCTCCACAT
BCl-2	B-cell lymphoma-2 like protein 4	NM_007527.3	F: CACCAGCTCTGAACAGATCATGA R: TCAGCCCATCTTCTTCCAGATGGT
IL-1β	Interleukin 1 beta, mRNA	NM_031512.2	F: CAGGATGAGGACCCAAGCAC R: GTCGTCATCATCCCACGAGT
NOS	Nitric oxide synthase 2	NM_012611.3	F: GGAGAAAACCCCAGGTGCTATT R: TCGATGGAGTCACATGCAGC

**Table 2:** Johnsen’s scores criteria for evaluation of spermatogenesis among studied groups.

Score	Definition and Criteria
10	Complete spermatogenesis with many mature spermatozoa
9	Many spermatozoa, with a disorganized germinal epithelium that showed sloughing into lumen
8	Presence of few spermatozoa (<5 to 10/seminiferous tubule)
7	Absence of spermatozoa, but many spermatids are present
6	Absence of spermatozoa, with few spermatids (<5/seminiferous tubule)
5	Absence of spermatozoa, and spermatid, with the presence of several spermatocytes
4	Absence of spermatozoa, and spermatid, with the presence of few spermatocytes (<5/seminiferous tubule)
3	Spermatogonia are the only cell present
2	Sertoli cells only present with absence of germ cells
1	No cells visualized in the tubular section

**Table 3:** List of antibodies, sources, working dilutions, and methods for antigen retrieval.

Antibody	Source	Dilution	Antigen Retrieval	Heating Condition
Rabbit polyclonal anti-active caspase 3	(#9662, Cell Signaling Technology, Danvers, MA, USA)	1:300	10 mM citrate buffer (pH 6.0)	105 °C, 20 min
Rabbit polyclonal anti-iNOS	(ab15323, Abcam, Cambridge, UK)	1:100	10 mM citrate buffer (pH 6.0)	105 °C, 20 min
Rabbit polyclonal anti-Iba1	(019-19741, Wako Osaka, Japan)	1:1200	10 mM citrate buffer (pH 6.0)	105 °C, 20 min
Rabbit polyclonal anti-MVH	(ab13840, Abcam, Cambridge, UK)	1:200	10 mM citrate buffer (pH 6.0)	105 °C, 20 min
Rabbit polyclonal anti-Sox9	(AB5535, Merck Millipore, Burlington, MA, USA)	1:1000	10 mM citrate buffer (pH 6.0)	105 °C, 20 min
Goat polyclonal anti-IL1β	(AB-401-NA, R&D Systems, Minneapolis, MN, USA)	1:200	10 mM citrate buffer (pH 6.0)	105 °C, 20 min
Goat polyclonal anti-PCNA	(sc-9857, Santa Cruz Biotechnology, Santa Cruz, CA, USA)	1:2000	Dako, 105 °C, 20 min	105 °C, 20 min
Rabbit polyclonal anti-inhibin alpha	(CM 171 A, Biocare Medical, Concord, CA, USA)	1:100	10 mM citrate buffer (pH 6.0)	105 °C, 20 min

**Table 4:** Pancreatic lipid peroxidation and antioxidant biomarkers, and serum testosterone in control and experiment groups and coefficients of variation (CV (%); intra- and inter- assay) values of tested biochemical and hormonal parameters.

Group	Pancreatic Tissue				Serum
	MDA (nmol/g)	NO (µmol/g)	SOD (U/g)	GPx (U/g)	Testosterone (ng/mL)
CTR	0.32 ± 0.03 ^c^	43.07 ± 1.03 ^b^	7.33 ± 0.28 ^b^	29.29 ± 1.02 ^ab^	7.51 + 0.27 ^a^
FIP	0.68 ± 0.03 ^a^	79.31 ± 0.89 ^a^	4.16 ± 0.08 ^c^	15.30 ± 0.47 ^c^	2.92 + 0.15 ^c^
CeNPs	0.36 ± 0.02 ^c^	32.32 ± 1.51 ^c^	7.57 ± 0.40 ^ab^	27.55 ± 0.59 ^b^	6.92 + 0.15 ^ab^
FIP+CeNPs	0.44 ± 0.001 ^b^	29.34 ± 0.56 ^c^	8.12 ± 0.23 ^a^	36.05 ± 2.24 ^a^	6.44 + 0.23 ^b^
Sig *p*-value	0.001	0.031	0.002	0.002	0.027
Intra-Assay	3.15	3.45	2.50	5.36	6.53
Inter-Assay	8.32	9.54	7.91	8.74	10.12

All values were expressed as mean ± SEM, *n* = 7. Values with different superscript letters within the same column are significantly different (*p* ≤ 0.05, one-way ANOVA, followed by Tukey’s HSD post hoc test for comparison). CTR: control, FIP: fipronil, CeNPs: cerium nanoparticles, MDA: malondialdehyde, NO: nitric oxide, SOD: superoxide dismutase, and GPx: glutathione peroxidase.

**Table 5:** The levels of relative mRNA expression of apoptotic genes (Casp3 and BCL2), anti-inflammatory genes (IL-1β and NOS), Ar, and IBP in the testes of rats.

Group	Casp3	BCL-2	IL-1β	NOS	Ar	IBP
CTR	1.00 ± 0.25 ^b^	1.00 ± 0.25 ^b^	1.00 ± 0.25 ^a^	1.00 ± 0.25 ^b^	1.00 ± 0.25 ^a^	1.00 ± 0.25 ^b^
FIP	1.8 ± 0.45 ^a^	0.04 ± 0.01 ^c^	1.53 ± 0.38 ^a^	1.50 ± 0.37 ^b^	0.36 ± 0.09 ^b^	0.28 ± 0.07 ^c^
CeNPs	0.78 ± 0.19 ^c^	1.25 ± 0.31 ^ab^	0.25 ± 0.06 ^b^	3.09 ± 0.77 ^a^	1.37 ± 0.34 ^a^	1.60 ± 0.40 ^ab^
FIP+CeNPs	0.08 ± 0.02 ^d^	1.90 ± 0.30 ^a^	0.01 ± 0.002 ^c^	0.019 ± 0.005 ^c^	1.21 ± 0.30 ^a^	1.96 ± 0.49 ^a^
Sig *p* value	0.002	0.001	0.005	0.003	0.027	0.038

All values were expressed as mean ± SEM, *n* = 7. Values with different superscript letters within the same column are significantly different (*p* ≤ 0.05, one-way ANOVA, followed by Tukey’s HSD post hoc test for comparison). CTR: control, FIP: fipronil, CeNPs: cerium nanoparticles, Casp3: caspase3, BCL2: cell lymphoma, IL-1β: interleukin 1β, NOS: nitric oxide synthase, Ar: androgen receptor, and IBP: inhibin B protein.

**Table 6:** Comparison of the Johnsen’s score among studied rat groups.

Groups	Johnsen’s Score
CTR	9.01 ± 0.54 ^b^
FIP	2.1 ± 0.29 ^a^
CeNPs	9.21 ± 0.73 ^b^
FIP+CeNPs	6.96 ± 0.24 ^b^

All values were expressed as mean ± SEM, *n* = 4. Values with different superscript letters (a & b) within the same column are significantly different (*p* ≤ 0.05, one-way ANOVA, followed by Tukey’s HSD post hoc test for comparison). CTR; control, FIP; fipronil, CeNPs; cerium nanoparticles.

## References

[1] Jensen T.K., Bonde J.P., Joffe M. (2006). The influence of occupational exposure on male reproductive function. Occup. Med..

[2] Wang X.-Z., Liu S.-S., Sun Y., Wu J.-Y., Zhou Y.-L., Zhang J.-H. (2009). Beta-cypermethrin impairs reproductive function in male mice by inducing oxidative stress. Theriogenology.

[3] Vidau C., Brunet J.-L., Badiou A., Belzunces L.P. (2009). Phenylpyrazole insecticides induce cytotoxicity by altering mechanisms involved in cellular energy supply in the human epithelial cell model Caco-2. Toxicol. In Vitro.

[4] Avudainayagam S., Megharaj M., Owens G., Kookana R.S., Chittleborough D., Naidu R. (2003). Chemistry of chromium in soils with emphasis on tannery waste sites. Reviews of Environmental Contamination and Toxicology.

[5] Gaines T.B. (1969). Acute toxicity of pesticides. Toxicol. Appl. Pharmacol..

[6] Kitulagodage M. (2011). Impact of Fipronil, a New Generation Pesticide, on Avian Development and Health. Ph.D. Thesis.

[7] World Health Organization (2010). The WHO Recommended Classification of Pesticides by Hazard and Guidelines to Classification 2009.

[8] Ohi M., Dalsenter P., Andrade A., Nascimento A. (2004). Reproductive adverse effects of fipronil in Wistar rats. Toxicol. Lett..

[9] Bencic D.C., Villeneuve D.L., Biales A.D., Blake L., Durhan E.J., Jensen K.M., Kahl M.D., Makynen E.A., Martinović-Weigelt D., Ankley G.T. (2013). Effects of the insecticide fipronil on reproductive endocrinology in the fathead minnow. Environ. Toxicol. Chem..

[10] Beshbishy A.M., Batiha G.E., Yokoyama N., Igarashi I. (2019). Ellagic acid microspheres restrict the growth of *Babesia* and *Theileria in vitro* and *Babesia microti in vivo*. Parasites Vectors.

[11] Das S., Dowding J.M., Klump K.E., McGinnis J.F., Self W., Seal S. (2013). Cerium oxide nanoparticles: Applications and prospects in nanomedicine. Nanomed. Nanotechnol. Biol. Med..

[12] Culcasi M., Benameur L., Mercier A., Lucchesi C., Rahmouni H., Asteian A., Casano G., Botta A., Kovacic H., Pietri S. (2012). EPR spin trapping evaluation of ROS production in human fibroblasts exposed to cerium oxide nanoparticles: Evidence for NADPH oxidase and mitochondrial stimulation. Chem. Biol. Interact..

[13] Korsvik C., Patil S., Seal S., Self W.T. (2007). Superoxide dismutase mimetic properties exhibited by vacancy engineered ceria nanoparticles. Chem. Commun..

[14] Celardo I., Pedersen J.Z., Traversa E., Ghibelli L. (2011). Pharmacological potential of cerium oxide nanoparticles. Nanoscale.

[15] Heckert E.G., Karakoti A.S., Seal S., Self W.T. (2008). The role of cerium redox state in the SOD mimetic activity of nanoceria. Biomaterials.

[16] Kobyliak N.M., Falalyeyeva T.M., Kuryk O.G., Beregova T.V., Bodnar P.M., Zholobak N.M., Shcherbakov O.B., Bubnov R.V., Spivak M.Y. (2015). Antioxidative effects of cerium dioxide nanoparticles ameliorate age-related male infertility: Optimistic results in rats and the review of clinical clues for integrative concept of men health and fertility. EPMA J..

[17] Caballero M., Ares I., Martinez M., Martinez-Larranaga M., Anadon A., Martinez M. (2015). Fipronil induces CYP isoforms in rats. Food Chem. Toxicol..

[18] Pakzad M., Fouladdel S., Nili-Ahmadabadi A., Pourkhalili N., Baeeri M., Azizi E., Sabzevari O., Ostad S.N., Abdollahi M. (2013). Sublethal exposures of diazinon alters glucose homostasis in Wistar rats: Biochemical and molecular evidences of oxidative stress in adipose tissues. Pestic. Biochem. Physiol..

[19] Draper H., Hadley M. (1990). Malondialdehyde determination as index of lipid Peroxidation. Methods in Enzymology.

[20] Davis K.L., Martin E., Turko I.V., Murad F. (2001). Novel effects of nitric oxide. Annu. Rev. Pharmacol. Toxicol..

[21] Nishikimi M., Appaji N., Yagi K. (1972). The occurrence of superoxide anion in the reaction of reduced phenazine methosulfate and molecular oxygen. Biochem. Biophys. Res. Commun..

[22] Paglia D.E., Valentine W.N. (1967). Studies on the quantitative and qualitative characterization of erythrocyte glutathione peroxidase. J. Lab. Clin. Med..

[23] Bancroft J.D., Layton C., Kim S. (2013). The hematoxylin and eosin, connective and mesenchymal tissues with their stains. Bancroft’s Theory and Practice of Histological Techniques.

[24] Layton C., Bancroft J.D., Kim S. (2013). Carbohydrates. Bancroft’s Theory and Practice of Histological Techniques.

[25] Gibson-Corley K.N., Olivier A.K., Meyerholz D.K. (2013). Principles for valid histopathologic scoring in research. Vet. Pathol..

[26] Johnsen S.G. (1970). Testicular biopsy score count—A method for registration of spermatogenesis in human testes: Normal values and results in 335 hypogonadal males. Horm. Res. Paediatr..

[27] Noreldin A.E., Elewa Y.H.A., Kon Y., Warita K., Hosaka Y.Z. (2018). Immunohistochemical localization of osteoblast activating peptide in the mouse kidney. Acta Histochem..

[28] Sysel A.M., Valli V.E., Nagle R.B., Bauer J.A. (2013). Immunohistochemical quantification of the vitamin B12 transport protein (TCII), cell surface receptor (TCII-R) and Ki-67 in human tumor xenografts. Anticancer Res..

[29] Vis A.N., Kranse R., Nigg A.L., van der Kwast T.H. (2000). Quantitative analysis of the decay of immunoreactivity in stored prostate needle biopsy sections. Am. J. Clin. Pathol..

[30] Ermer J., Ermer J., Miller J.H.M. (2005). Method validation in pharmaceutical analysis. MS Applications in Drug Development Reference Materials for Chemical Analysis.

[31] International Programme on Chemical Safety (IPCS) (2014). Harmonization Project Document 11: Guidance Document on Evaluating And Expressing Uncertainty in Hazard Characterization.

[32] Badgujar P.C., Pawar N.N., Chandratre G.A., Telang A., Sharma A. (2015). Fipronil induced oxidative stress in kidney and brain of mice: Protective effect of vitamin E and vitamin C. Pestic. Biochem. Physiol..

[33] Abdelaziz A.S., Kamel M.A., Ahmed A.I., Shalaby S.I., El-Darier S.M., Magdy Beshbishy A., Batiha G.E., Alomar S.Y., Khodeer D.M. (2020). Chemotherapeutic potential of *Epimedium brevicornum* extract: The cGMP-specific PDE5 inhibitor as anti-infertility agent following long-term administration of tramadol in male rats. Antibiotics.

[34] Circu M.L., Aw T.Y. (2010). Reactive oxygen species, cellular redox systems, and apoptosis. Free Radic. Biol. Med..

[35] Pierini D., Bryan N.S. (2015). Nitric oxide availability as a marker of oxidative stress. Advanced Protocols in Oxidative Stress III.

[36] Sen S., Chakraborty R. (2011). The role of antioxidants in human health. Oxidative Stress: Diagnostics, Prevention, and Therapy.

[37] Bhattacharjee R., Sil P.C. (2006). The protein fraction of *Phyllanthus niruri* plays a protective role against acetaminophen induced hepatic disorder via its antioxidant properties. Phytother. Res..

[38] Kavlock R., Cummings A. (2005). Mode of action: Inhibition of androgen receptor function—Vinclozolin-induced malformations in reproductive development. Crit. Rev. Toxicol..

[39] Haider S.G. (2004). Cell biology of Leydig cells in the testis. Int. Rev. Cytol..

[40] Kasahara E., Miyoshi M., Konaka R., Hiramoto K., Sasaki J., Tokuda M., Nakano Y., Inoue M. (2002). Role of oxidative stress in germ cell apoptosis induced by di (2-ethylhexyl) phthalate. Biochem. J..

[41] Gao H.-B., Tong M.-H., Hu Y.-Q., You H.-Y., Guo Q.-S., Ge R.-S., Hardy M.P. (2003). Mechanisms of glucocorticoid-induced Leydig cell apoptosis. Mol. Cell. Endocrinol..

[42] Khan S., Jan M., Kumar D., Telang A. (2015). Firpronil induced spermotoxicity is associated with oxidative stress, DNA damage and apoptosis in male rats. Pestic. Biochem. Physiol..

[43] Skommer J., Wlodkowic D., Deptala A. (2007). Larger than life: Mitochondria and the Bcl-2 family. Leuk. Res..

[44] Sen N., Das B., Ganguly A., Mukherjee T., Tripathi G., Bandyopadhyay S., Rakshit S., Sen T., Majumder H. (2004). Camptothecin induced mitochondrial dysfunction leading to programmed cell death in unicellular hemoflagellate Leishmania donovani. Cell Death Differ..

[45] Puka-Sundvall M., Gajkowska B., Cholewinski M., Blomgren K., Lazarewicz J.W., Hagberg H. (2000). Subcellular distribution of calcium and ultrastructural changes after cerebral hypoxia-ischemia in immature rats. Dev. Brain Res..

[46] Chan W.Y., Lorke D.E., Tiu S.C., Yew D.T. (2002). Proliferation and apoptosis in the developing human neocortex. Anat. Rec..

[47] Sharpe R., Turner K., McKinnell C., Groome N., Atanassova N., Millar M., Buchanan D., Cooke P.S. (1999). Inhibin B levels in plasma of the male rat from birth to adulthood: Effect of experimental manipulation of Sertoli cell number. J. Androl..

[48] Meachem S., Nieschlag E., Simoni M. (2001). Inhibin B in male reproduction: Pathophysiology and clinical relevance. Eur. J. Endocrinol..

[49] Adeyemi O.S., Shittu E.O., Akpor O.B., Rotimi D., Batiha G.E. (2020). Silver nanoparticles restrict microbial growth by promoting oxidative stress and DNA damage. EXCLI J..

[50] Lambright C., Ostby J., Bobseine K., Wilson V., Hotchkiss A., Mann P., Gray L. (2000). Cellular and molecular mechanisms of action of linuron: An antiandrogenic herbicide that produces reproductive malformations in male rats. Toxicol. Sci..

[51] Saradha B., Mathur P. (2006). Effect of environmental contaminants on male reproduction. Environ. Toxicol. Pharmacol..

[52] Zeng L., Kong X.-T., Su J.-W., Xia T.-L., Na Y.-Q., Guo Y.-L. (2001). Evaluation of germ-cell kinetics in infertile patients with proliferating cell nuclear antigen proliferating index. Asian J. Androl..

[53] Yamada M., Ohsawa K., Imai Y., Kohsaka S., Kamitori S. (2006). X-ray structures of the microglia/macrophage-specific protein Iba1 from human and mouse demonstrate novel molecular conformation change induced by calcium binding. J. Mol. Biol..

[54] Tanaka S., Suzuki K., Watanabe M., Matsuda A., Tone S., Koike T. (1998). Upregulation of a new microglial gene, mrf-1, in response to programmed neuronal cell death and degeneration. J. Neurosci..

[55] Utans U., Arceci R.J., Yamashita Y., Russell M.E. (1995). Cloning and characterization of allograft inflammatory factor-1: A novel macrophage factor identified in rat cardiac allografts with chronic rejection. J. Clin. Investig..

[56] Chen Z.-W., Ahren B., Östenson C.-G., Cintra A., Bergman T., Möller C., Fuxe K., Mutt V., Jörnvall H., Efendic S. (1997). Identification, isolation, and characterization of daintain (allograft inflammatory factor 1), a macrophage polypeptide with effects on insulin secretion and abundantly present in the pancreas of prediabetic BB rats. Proc. Natl. Acad. Sci. USA.

[57] Ito D., Imai Y., Ohsawa K., Nakajima K., Fukuuchi Y., Kohsaka S. (1998). Microglia-specific localisation of a novel calcium binding protein, Iba1. Mol. Brain Res..

[58] Iida H., Doiguchi M., Yamashita H., Sugimachi S., Ichinose J., Mori T., Shibata Y. (2001). Spermatid-specific expression of Iba1, an ionized calcium binding adapter molecule-1, in rat testis. Biol. Reprod..

[59] Sasaki Y., Ohsawa K., Kanazawa H., Kohsaka S., Imai Y. (2001). Iba1 is an actin-cross-linking protein in macrophages/microglia. Biochem. Biophys. Res. Commun..

[60] Ohsawa K., Imai Y., Kanazawa H., Sasaki Y., Kohsaka S. (2000). Involvement of Iba1 in membrane ruffling and phagocytosis of macrophages/microglia. J. Cell Sci..

[61] Wilson M.J., Jeyasuria P., Parker K.L., Koopman P. (2005). The transcription factors steroidogenic factor-1 and SOX9 regulate expression of Vanin-1 during mouse testis development. J. Biol. Chem..

[62] Toyooka Y., Tsunekawa N., Takahashi Y., Matsui Y., Satoh M., Noce T. (2000). Expression and intracellular localization of mouse Vasa-homologue protein during germ cell development. Mech. Dev..

[63] Nassar A., Salim Y., Eid K., Shaheen H.M., Saati A.A., Hetta H.F., Elmistekawy A., Batiha G.E. (2020). Ameliorative effects of honey, propolis, pollen, and royal jelly mixture against chronic toxicity of sumithion insecticide in white albino rats. Molecules.

